# The vagal ganglia transcriptome identifies candidate therapeutics for airway hyperreactivity

**DOI:** 10.1152/ajplung.00557.2017

**Published:** 2018-04-05

**Authors:** Leah R. Reznikov, David K. Meyerholz, Mahmoud Abou Alaiwa, Shin-Ping Kuan, Yan-Shin J. Liao, Nicholas L. Bormann, Thomas B. Bair, Margaret Price, David A. Stoltz, Michael J. Welsh

**Affiliations:** ^1^Department of Physiological Sciences, University of Florida, Gainesville, Florida; ^2^Department of Pathology, University of Iowa, Iowa City, Iowa; ^3^Department of Internal Medicine, University of Iowa, Iowa City, Iowa; ^4^Iowa Institute of Human Genetics, University of Iowa, Iowa City, Iowa; ^5^Molecular Physiology and Biophysics, University of Iowa, Iowa City, Iowa; ^6^Pappajohn Biomedical Institute, Roy J. and Lucille A. Carver College of Medicine, University of Iowa, Iowa City, Iowa; ^7^Department of Biomedical Engineering, College of Engineering, University of Iowa, Iowa City, Iowa; ^8^Howard Hughes Medical Institute, University of Iowa, Iowa City, Iowa

**Keywords:** airway hyperreactivity, asthma, neurons, therapeutics, transcriptome sequencing, vagus

## Abstract

Mainstay therapeutics are ineffective in some people with asthma, suggesting a need for additional agents. In the current study, we used vagal ganglia transcriptome profiling and connectivity mapping to identify compounds beneficial for alleviating airway hyperreactivity (AHR). As a comparison, we also used previously published transcriptome data from sensitized mouse lungs and human asthmatic endobronchial biopsies. All transcriptomes revealed agents beneficial for mitigating AHR; however, only the vagal ganglia transcriptome identified agents used clinically to treat asthma (flunisolide, isoetarine). We also tested one compound identified by vagal ganglia transcriptome profiling that had not previously been linked to asthma and found that it had bronchodilator effects in both mouse and pig airways. These data suggest that transcriptome profiling of the vagal ganglia might be a novel strategy to identify potential asthma therapeutics.

## INTRODUCTION

Asthma is a chronic airway disease characterized by wheezing, chest tightness, cough, and variable airflow obstruction (31). Exaggerated airway narrowing in response to a variety of stimuli, termed airway hyperreactivity (AHR), is a hallmark feature of asthma (13). Our current understanding of AHR places inflammation at the center of its pathogenesis (34). However, therapeutics that directly target inflammation, such as glucocorticoids, are not effective in all people with asthma (65). Thus the identification and development of additional therapeutics that do not directly target inflammatory pathways might be of significant clinical value.

One attractive candidate is the nervous system and specifically, the vagus nerve. As early as the 17th century, anticholinergics, which prevent the postsynaptic actions of acetylcholine released from the vagus nerve, were explored as therapeutics for asthma (64). However, the contribution of the nervous system to AHR is complex and involves inflammation-dependent and -independent mechanisms. Specifically, Tränkner and colleagues (91) found that ablation of vagal sensory neurons expressing the *transient receptor potential vanilloid 1*, prevented AHR in ovalbumin (OVA)-sensitized mice without decreasing airway inflammation. In our previous work (66), we found that loss of *acid-sensing ion channel* (*ASIC*) *1a*, a proton-gated neuronal cation channel expressed in the vagal ganglia and other neural compartments, also prevented AHR without decreasing airway inflammation.

Yet others have found that the silencing of vagal sensory neurons that express Nav1.8 (88) or elimination of the *transient receptor potential cation channel, subfamily A, member 1*, decreases both AHR and airway inflammation (10). Thus two contrasting roles for vagal sensory neurons have been described: those that modify AHR, independent of inflammation, and those that modify AHR and inflammation. This observation makes vagal ganglion neurons an appealing target for therapeutics.

Molecular profiling has enhanced therapeutic discovery for several diseases (17, 40, 81). In light of this and in light of our recent observation that mice with targeted disruptions to *ASIC1a* lack AHR despite robust inflammation (66), we hypothesized that vagal ganglia transcriptome profiling might provide unique insight into potential asthma therapeutics. To test this hypothesis, we probed the vagal ganglia of nonsensitized and OVA-sensitized wild-type (WT) and *ASIC1a^−/−^* mice. This enabled the comparison of transcripts from animals that lacked AHR but exhibited inflammation (OVA-sensitized *ASIC1a^−/−^* mice) with transcripts from animals that displayed both inflammation and AHR (OVA-sensitized WT mice) (66). We also used previously published transcriptome data of OVA-sensitized murine lung tissue (12) and endobronchial biopsies of human asthmatics (100) as a comparison and control.

## MATERIALS AND METHODS

### 

#### Animals.

Adult (8–9 wk old) male A*SIC1a^−/−^* (62) and WT mice were maintained on a congenic C57BL/6J background. Additional C57BL/6 WT mice were obtained from Charles River Laboratories (Wilmington, MA) and were used only for testing acute effects of alverine citrate and for making lung slices. Male piglets (2–3 days old) were euthanized by intracardiac Euthasol (Vibrac, Fort Worth, TX), as previously described (85). All procedures adhered to and were approved by the University of Iowa Animal Care and Use Committee.

#### OVA sensitization.

Mice were sensitized, as previously described (66, 72). Briefly, 8- to 9-wk-old male mice were sensitized by intraperitoneal injection of 10 μg OVA (MilliporeSigma, St. Louis, MO), mixed with 1 mg alum in 0.9% saline on *days 0* and *7*. Control mice received saline with 1 mg alum on *days 0* and *7*. Starting on *day 14*, a 1% OVA or 0.9% saline solution was nebulized for 40 min to the mice for 3 consecutive days. This equated to each mouse seeing three total challenges, with a single challenge occurring each day for 3 consecutive days (*days 14–16*).

For testing the effect of alverine citrate during the OVA-sensitization protocol, alverine citrate was provided as a multiple dose regimen. Specifically, alverine citrate or 0.5% DMSO vehicle was dissolved in the alum/OVA mixture at dose 15 mg/kg and delivered at *days 0* and *7*. On *days 14–16*, alverine citrate or 0.5% DMSO vehicle was delivered intraperitoneally in 0.9% saline, 30 min before nebulization with 1% OVA. On the day of flexiVent measurements (*day 17*), alverine citrate or 0.5% DMSO was delivered intraperitoneally in 0.9% saline, 30 min before testing by flexiVent.

#### Bronchoalveolar lavage and analyses.

Lungs received three sequential, 1-ml lavages of 0.9% sterile saline delivered into the airways through a cannula secured in the euthanized mouse trachea, as previously described (66). All collected material from one mouse was pooled and spun at 500 *g*, and the supernatant was removed and frozen at −80°C. Cells were counted on a hemocytometer, as previously described (66). IL-4, IL-5, and IL-13 were assayed by DuoSet ELISA kits (R&D Systems, Minneapolis, MN).

#### Vagal ganglia isolation.

Mice were euthanized by overdose of isoflurane inhalation following the OVA-sensitization protocol (on *day 17* of protocol). Mice used for vagal ganglia collection did not undergo flexiVent procedures because of the potential confound that methacholine and mechanical stimulation of the airways (i.e., methacholine-induced bronchoconstriction) might impart on the vagal ganglia transcriptome. This approach is consistent with previous studies in which transcriptome sequencing has been performed in OVA-sensitized mouse lung tissues without analysis of airway resistance (12). Following euthanasia, the vagal ganglia were exposed by gently tracing the vagus nerve back to the base of the skull using protocols similar to those previously described (50, 66). The ganglia were delicately removed and immediately placed in TRIzol stored at −80°C until RNA isolation. The total ganglia, including any connective tissues of the ganglion capsule, inflammatory cells that may have migrated to the ganglion, satellite cells, blood cells, and blood vessels covering the ganglia, were not removed and were therefore included.

#### RNA isolation and quantitative RT-PCR.

RNA from the vagal ganglia was isolated using the RNeasy Lipid Tissue kit (a chloroform/phenol-based RNA extraction kit; Qiagen, Germantown, MD), according to the manufacturer’s instructions. The optional DNase digestion was performed according to the manufacturer’s instructions using an RNase-free DNase set (Qiagen). RNA integrity was assessed with an Agilent bioanalyzer by the University of Iowa DNA Core. To confirm RNA sequencing results, RNA was reverse transcribed using Superscript VILO Master Mix (Thermo Fisher Scientific, Waltham, MA), according to the manufacturer’s instructions. Briefly, the RNA and Master Mix were allowed to incubate for 10 min at 25°C, followed by 60 min at 42°C, followed by 5 min at 85°C. Primer and probes for murine *peroxisome proliferator-activated receptor* (*ppar*)*γc1A*, *cluster of differentiation* (*CD*)*93*, *chondrolectin* (*chodl*), and *actin* were obtained from GeneCopoeia (Rockville, MD) and run on a 7500 Fast Real-Time PCR System (Thermo Fisher Scientific) following the manufacturers’ protocol. *pparγc1A*, a gene identified by *signature 1*, was chosen because of the proposed bronchodilatory effect of PPARγ1 agonists (82). *CD93*, a gene that increased in response to OVA sensitization in *ASIC1a^−/−^* mice, compared with nonsensitized *ASIC1a^−/−^* mice, was chosen because it is reportedly involved in nervous system inflammation (47). *Chodl*, a gene that showed differential regulation in OVA-sensitized *ASIC1a^−/−^* and OVA-sensitized WT mice, was chosen because it affects neuronal outgrowth and survival (79).

RNA from total mouse lung was isolated using the RNeasy Lipid Tissue kit (according to the manufacturer’s instructions. The optional DNase digestion was also Qiagen), performed using a RNase-free DNase set (Qiagen), as detailed by the manufacturer’s instructions. RNA integrity was assessed by an Agilent nioanalyzer by the University of Iowa DNA Core. The lung RNA was reverse transcribed using Superscript VILO Master Mix (Thermo Fisher Scientific), according to the manufacturer’s instructions. Briefly, the RNA and Master Mix were allowed to incubate for 10 min at 25°C, followed by 60 min at 42°C, followed by 5 min at 85°C. Quantitative PCR for *mucin 5AC* (*muc5AC*) was performed using Fast SYBR Green Master Mix (Qiagen) and run on a 7500 Fast Real-Time PCR System (Thermo Fisher Scientific), following the manufacturer’s protocol. Primers were designed for murine *muc5AC*, as previously described (66, 72): *muc5ac* forward 5′-GTGGTGGAAACTGACATTGG-3′; *muc5ac* reverse 5′-CATCAAAGTTCCCACACAGG-3′. Primers for mouse *actin* were used as a housekeeping gene: *actin* forward 5′-CTGTGGCATCCATGAAACTACA-3′; actin reverse 5′-GTAATCTCCTTCTGCATCCTGTCA-3′.

#### flexiVent.

flexiVent procedures were performed as previously described (66, 72). Briefly, a tracheotomy was performed in anesthetized mice (ketamine-xylazine), and a cannula (blunted 18-gauge needle) was inserted into the tracheas. Mice were ventilated at 150 breaths/min at a volume of 10 ml/kg body mass and then administered a paralytic (rocuronium bromide). Increasing doses of methacholine were aerosolized using an ultrasonic nebulizer. The aerosols were delivered for 10 s into the inspiratory line of the ventilator. Measurements for each methacholine dose were taken at 10-s intervals over the course of 5 min. Airway resistance was measured using a single compartment model (i.e., dynamic resistance of the respiratory system). For drug studies, alverine citrate or vehicle was delivered at designated doses, 30 min before the start of flexiVent measurements.

#### Chemicals.

Alverine citrate (Selleckchem.com, Houston, TX) was dissolved in 0.5% DMSO/0.9% saline solution at a dose of 15 mg/ml. The pH of nebulized alverine citrate was titrated with NaOH to equal the pH of 0.9% saline (pH ~5.5). Substance P (MilliporeSigma) was dissolved in PBS, not containing calcium or magnesium ions (PBS^−/−^). Acetyl-β-methacholine-chloride (MilliporeSigma) was dissolved in 0.9% saline for flexiVent studies or PBS^−/−^ for lung slice studies.

#### Histopathology.

Following mouse euthanasia, the left lung was removed and placed in 10% normal-buffered formalin. Samples were sectioned and stained as previously described (51, 66). A pathologist masked to groups performed scoring on hematoxylin-eosin-stained mouse lung sections (30). The following scores were assigned for bronchoperivascular inflammation severity: 1, within normal limits; 2, focal solitary cells with uncommon aggregates; 3, multifocal nominal- to moderate-sized aggregates; and 4, moderate to high cellularity, multifocal, large cellular aggregates that may be expansive into adjacent tissues. The following scores were assigned for perivascular inflammation distribution: 1, within normal limits; 2, minor to localized aggregates, <33% of lung; 3, multifocal aggregates, 33–66% of lung; 4, coalescing to widespread, >66% of lung.

#### RNA sequencing.

The vagal ganglia cDNA library construction and RNA sequencing were performed by the University of Iowa DNA Core. Briefly, 5 ng total RNA was used to prepare cDNA using the Ovation RNA-Seq System V2 (NuGen Technologies, San Carlos, CA), following the manufacturer’s protocol. The resulting cDNA was sheared using the Covaris E220-focused ultrasonicator (Covaris, Woburn, MA), such that the majority of the fragments were in the range of 150–500 bp. Sheared cDNA (500 ng) was taken into the KAPA Hyper Prep kit for Illumina sequencing (KAPA Biosystems, Wilmington, MA), and the indexed sequencing libraries generated were pooled in equimolar concentrations. The pooled libraries were sequenced on the HiSeq Sequencer 2000 (Illumina, San Diego, CA) with a 100-bp paired-end sequencing by synthesis chemistry. The vagal ganglia of three individual mice for each condition were prepared separately and used.

#### Mapping and differential gene expression.

Fastq files were aligned to the mouse mm9 genome using tophat2 (v. 2.0.13). Aligned files were summarized to counts via the featureCount tool, part of the subread package (v. 1.4.6). Count files were imported into R, and differential expression was assessed using the DESeq2 package (v. 1.5.3). We considered all transcript changes with *P* ≤ 0.05 as significant.

#### Lung slices.

Mouse lungs were excised and filled with a 2% low-melt agarose/DMEM solution by cannulating the trachea with a 22-gauge needle. For porcine lung slices, the porpoise lobe was removed and inflated with 2% low-melt agarose, as previously described (18). Both murine and porcine lungs were sectioned on a sliding microtome at a thickness of 300 µm. Slices were cultured in 90% DMEM-10% FBS-1% penicillin-streptavidin for 2 days. They were imaged with an Olympus X81 inverted microscope. Time-lapse video was taken with images captured every 5 s.

#### CMAP Build 02.

Transcripts were converted to Affymetrix U133A identifiers when identifiers were available (*signature 1*: 11/15, 31/44; *signature 2*: 318/448, 83/136; *signature 3*: 90/139, 4/9; *signature 4*: 19/28, 12/18). The connectivity map (CMAP) was queried and compound lists obtained using the “detailed results” function. Compound classifications were assigned using DrugCentral (95), ChEMBL (56), and PubChem (75).

#### Literature mining.

The name of each compound and the word “asthma” were searched in PubMed. Titles of articles were used for initial screening; a clear relevance to asthma or AHR was needed to merit further screening. Key words for title screening included airway, allergic, sensitization, disease, bronchoconstriction, hyperreactivity, hyperresponsiveness, and asthma. Abstracts and articles that were selected based on title were read. Attention was paid to whether a compound provided benefit in asthma, AHR, or bronchoconstriction. In cases where there were greater than three studies, more than two studies needed to show a negative effect for a compound to be deemed as having a worsening effect. In cases where there were greater than three studies, more than two studies needed to show a beneficial effect for the compound to be deemed as having a beneficial effect.

#### DisGeNET.

The list of transcripts comprising *signatures 1–3* was individually queried using the “gene” database. The diseases associated with each individual transcript were downloaded in Excel and compiled into a single worksheet.

#### Statistical analysis.

A two-way ANOVA was performed for studies with two or more groups and two or more treatments. When two or more groups were present, but only one condition was being tested, a one-way ANOVA was performed. Post hoc comparisons were performed using a Sidak’s multiple comparison test or least significant difference test. For RNA sequencing, only two treatments were compared at a time using DeSeq2 analysis in R. For histopathological scoring, a nonparametric Mann-Whitney test was performed. Contingency tables and χ^2^ tests or Fisher’s exact test were used to examine the dependency of a compound being beneficial or negative on the signature being queried. For all other studies, a Student’s unpaired *t*-test was performed. All statistical analysis was performed using GraphPad Prism 7.0a. Significance for all tests was assessed as *P* < 0.05.

## RESULTS

### 

#### Loss of ASIC1a and OVA sensitization influence the vagal ganglia transcriptome.

**Fig. 1.**
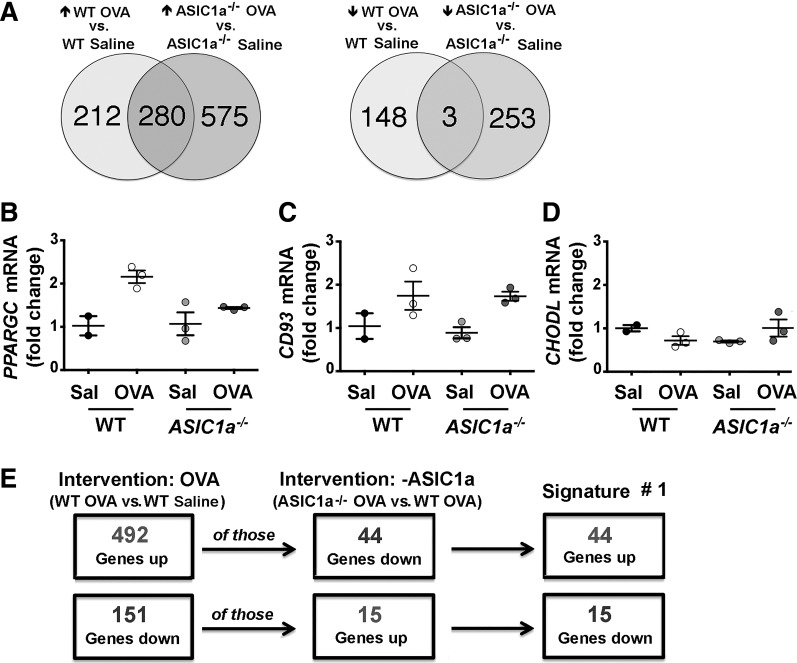
Ovalbumin (OVA)-responsive transcripts in the vagal ganglia of wild-type (WT) and acid-sensing ion channel (ASIC)1a^−/−^ mice. *A*: Venn diagram showing numbers of transcripts that were increased or decreased by OVA sensitization in WT (*left*) or *ASIC1a^−/−^* (*right*) mice. Data are from RNA sequencing of the vagal ganglia of 3 individual mice for each condition. Quantitative RT-PCR was used to validate transcript changes identified by RNA sequencing for *peroxisome proliferator-activated receptor* (*PPARGC; B*), *cluster of differentiation* (*CD*)*93* (*C*), and *chondrolectin* (*CHODL*; *D*). Data are expressed as means ± SE and are relative to their respective nonsensitized control. Individual points are data collected from a single mouse. *E*: flow chart depicting the strategy to obtain *signature 1*. Sal, saline (nonsensitized).

OVA sensitization is a common model that induces AHR in laboratory animals (58). Therefore, we first evaluated the vagal ganglia transcriptional response to OVA sensitization. We found that OVA sensitization increased 492 transcripts and decreased 151 transcripts in the vagal ganglia of WT mice compared with nonsensitized WT mice (Fig. 1*A* and Supplemental Table S1). By contrast, in *ASIC1a^−/−^* mice, OVA sensitization increased 855 transcripts and decreased 256 transcripts compared with nonsensitized *ASIC1a^−/−^* mice (Fig. 1*A* and Supplemental Table S1). Because previous studies have shown that the disruption of the *ASIC1a* gene or inhibition of ASIC currents changes neural activity (102, 103), we also examined the effect of loss of ASIC1a on the vagal ganglion transcriptome in nonsensitized mice. We found that loss of ASIC1a increased 159 transcripts and decreased 269 transcripts in the vagal ganglia compared with WT mice (Supplemental Table S2). The use of primer and probe sets for a few select transcripts, with our limited RNA remaining, revealed transcript changes consistent with those found by RNA sequencing (Fig. 1, *B*–*D*). Thus OVA sensitization impacted the vagal ganglia transcriptomes in both WT and *ASIC1a^−/−^* mice. Moreover, the presence or absence of ASIC1a influenced the vagal ganglia transcriptional response to OVA sensitization.

#### A portion of OVA-responsive vagal ganglion transcripts are normalized by loss of ASIC1a and offer potential therapeutic value.

We next implemented a nonconventional analysis akin to identifying transcripts that were increased or decreased in a disease state (i.e., OVA sensitization) but reversed following an intervention that elicited benefit (i.e., loss of ASIC1a). That is, we asked whether any of the 492 transcripts elevated by OVA sensitization in WT mice were decreased or “normalized” by loss of ASIC1a; we identified 44 transcripts (Fig. 1*E* and Supplemental Table S1). We also asked whether any of the 151 transcripts repressed by OVA sensitization in WT mice were increased or normalized by loss of ASIC1a; we identified 15 transcripts (Fig. 1*E* and Supplemental Table S1). We termed this list of 59 (i.e., 44 + 15) transcripts *signature 1*.

We predicted that some transcripts comprising *signature 1* might be of therapeutic significance. For example, if a transcript played a causal role in the manifestation of AHR, then its expression might appear normalized by loss of ASIC1a (i.e., *ASIC1a^−/−^* mice lack AHR) (66). Alternatively, if a transcript’s expression was induced or suppressed as part of a compensatory and/or adaptive response to AHR, then its expression might also appear normalized by loss of ASIC1a, since ASIC1a^−/−^ mice lack AHR (66). In the latter case, such transcripts might be beneficial and/or have protective effects (38, 78, 84). We also conceived that *signature 1* transcripts might have no bearing or role in AHR.

**Fig. 2.**
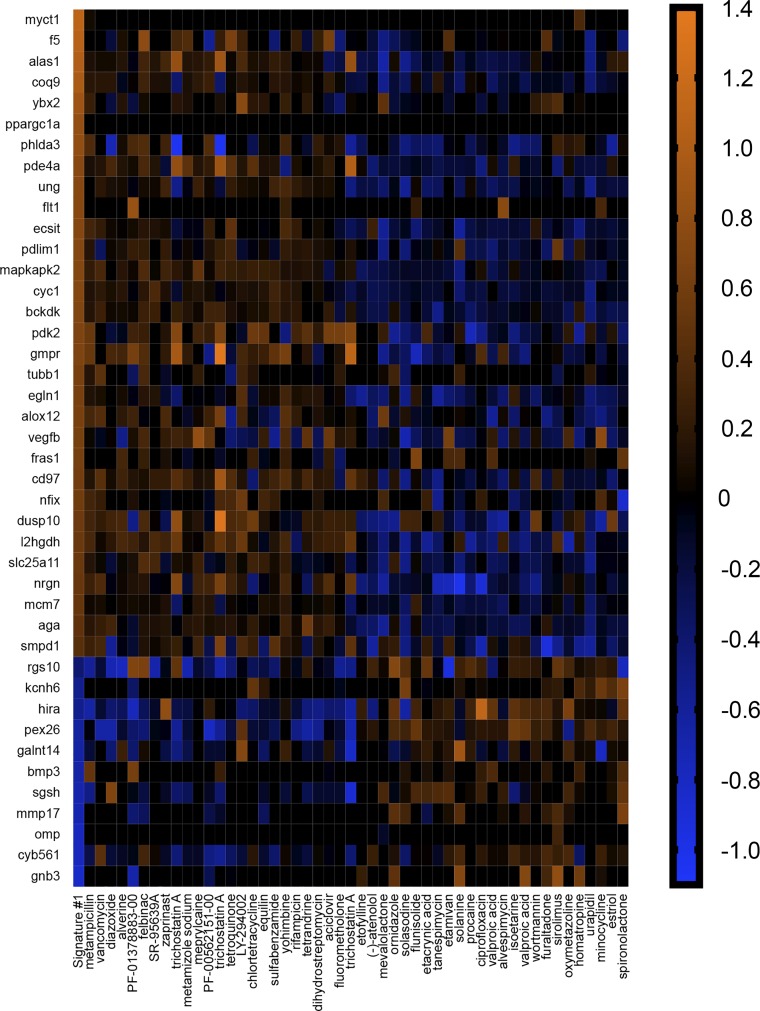
*Signature 1* identifies agents useful for treating airway hyperreactivity. Heatmap illustrating the log2 fold changes of transcripts comprising *signature 1* and associated compounds inducing the same (left-most 25 compounds) or opposite (right-most 25 compounds) gene signatures. Compounds were obtained through query of the connectivity map (CMAP). Genes (11/15 and 31/44) had Affymetrix identifiers that could be used in CMAP. Original scale is in log2 fold change.

**Fig. 3.**
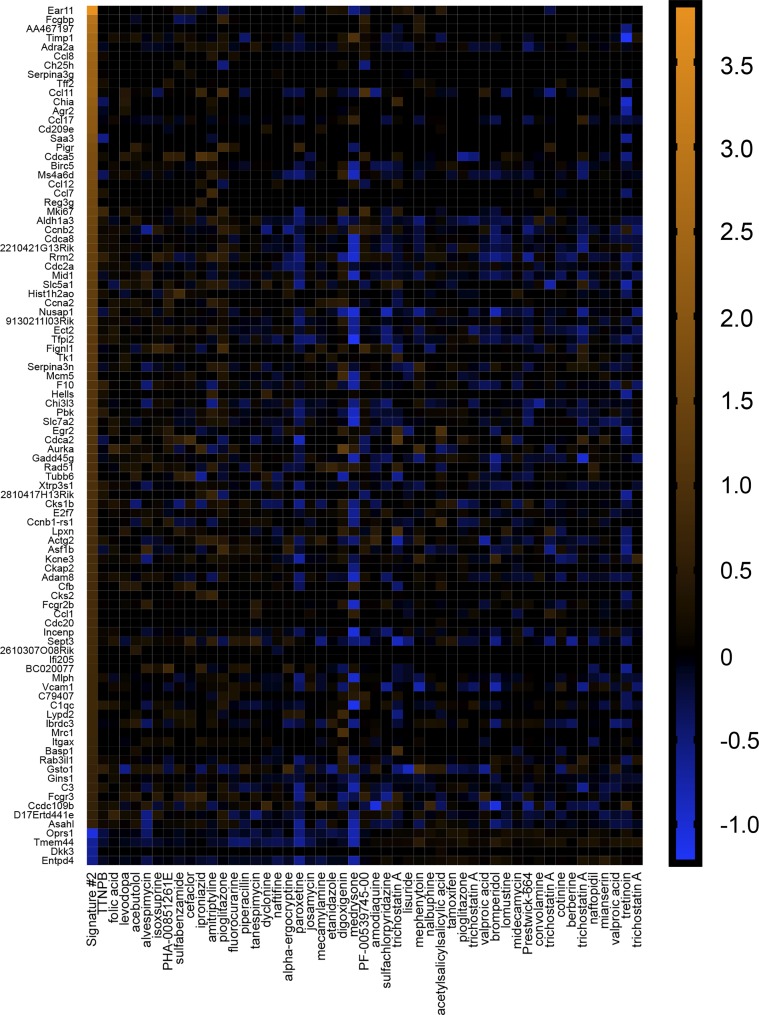
*Signature 2* identifies agents useful for treating airway hyperreactivity. Heatmap illustrating the log2 fold changes of transcripts comprising *signature 2* and associated compounds inducing the same (left-most 25 compounds) or opposite (right-most 25 compounds) gene signatures. Compounds were obtained through query of the connectivity map (CMAP). Genes (90/139 and 4/9) had Affymetrix identifiers that could be used in CMAP. Original scale is in log2 fold change.

**Fig. 4.**
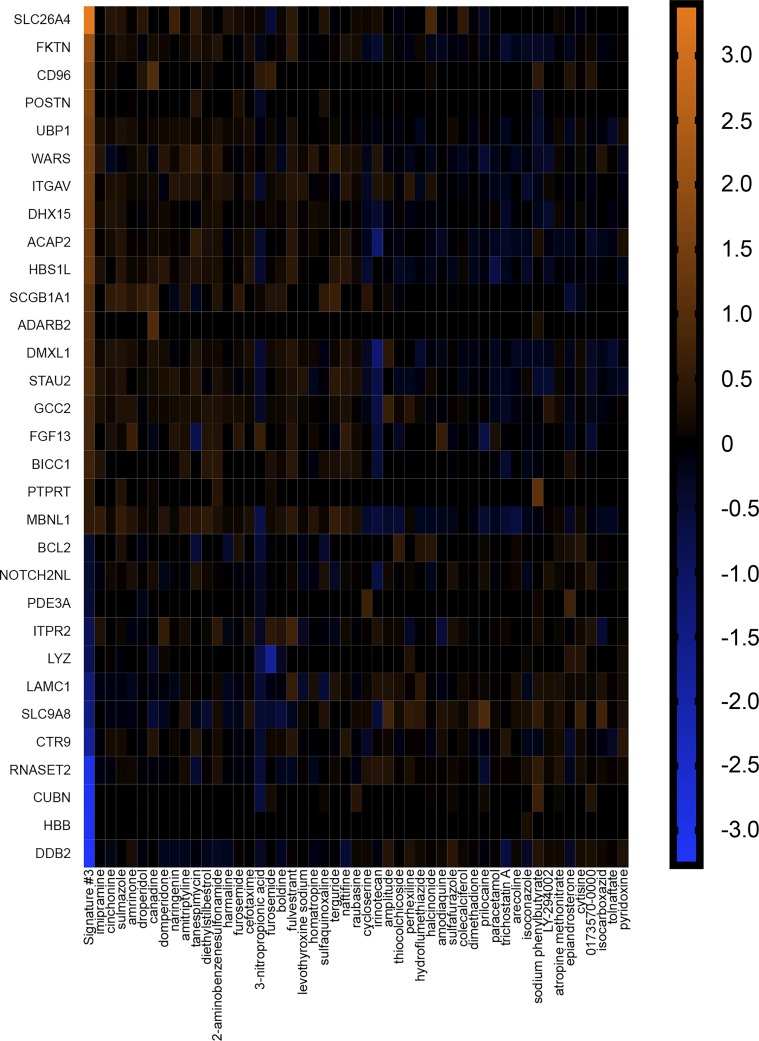
*Signature 3* identifies agents useful for treating airway hyperreactivity. Heatmap illustrating the log2 fold changes of transcripts comprising *signature 3* and associated compounds inducing the same (left-most 25 compounds) or opposite (right-most 25 compounds) gene signatures. Compounds were obtained through query of the connectivity map (CMAP). Genes (19/28 and 12/18) had Affymetrix identifiers that could be used in CMAP. Original scale is in log2 fold change.

To explore these possibilities, we performed connectivity mapping. The CMAP is a database of gene signatures derived from 6,100 compounds applied to human cell lines (41). It provides information regarding the connectedness of genes, drugs, and disease states through identification of common gene-expression signatures (86). If *signature 1* transcripts participated in the manifestation of AHR, then compounds with gene signatures that mimicked *signature 1* might induce AHR or worsen asthma symptoms (Fig. 2). The converse might also be true, in which compounds with gene signatures that reversed *signature 1* might be beneficial and mitigate AHR or alleviate asthma symptoms. On the other hand, if transcripts represented protective, beneficial adaptations secondary to AHR, then compounds with gene signatures that mimicked *signature 1* might be beneficial. If true, then compounds with gene signatures that reversed *signature 1* might also be beneficial or at least not be of negative consequences. For example, imagine a gene with a baseline expression of one. In response to inflammation, it increases its expression to 10 as means to compensate and/or protect against inflammation. If reversed back to one, a negative consequence would not be expected, as that was the resting (baseline) expression. Therefore, if transcripts comprising *signature 1* were beneficial due to compensatory changes, then compounds that reversed *signature 1* would not be of anticipated negative consequence, since reversal would effectively be returning to a resting state. As a comparison, we also probed CMAP with transcriptome data of OVA-sensitized murine lung tissue (12) (*signature 2*; Fig. 3 and Supplemental Table S3) and endobronchial biopsies of human asthmatics (100) (*signature 3*; Fig. 4 and Supplemental Table S3).

We first looked at compounds that mimicked each signature. We found that three of the top 25 (vancomycin, metamizole sodium, and rifampicin) that mimicked *signature 1* (Fig. 2) worsened asthma symptoms in people (14, 21, 39) (Table 1), whereas seven were beneficial and decreased airway obstruction (57), smooth muscle contraction (6, 99), and AHR (3, 22) (Table 1). For *signature 2* (Fig. 3), two compounds were of negative consequence and increased risk (7) or induced (52) asthma, whereas four were beneficial and decreased bronchoconstriction (2, 5, 28) or AHR (55) (Table 2). Similar results were observed with *signature 3* (Fig. 4), in which two compounds were of negative consequence and increased risk (97) or induced (9) asthma, and eight were of benefit and decreased bronchoconstriction (2, 44, 48, 60, 73) or AHR (37, 76) (Table 3). One compound that mimicked *signature 2*—isoxsuprine—had a single report, suggesting that it decreased bronchoconstriction in asthma (93), and an additional single report, suggesting its use was a risk factor for the development of asthma (11). A 3 × 2 contingency table analysis and a two-tailed χ^2^ test revealed that the chance of identification of a compound that was of beneficial consequence in asthma/AHR was not different among signatures [χ^2^ (2, *n* = 75) = 1.83, *P* = 0.41].

**Table 1.** CMAP compounds that mimicked signature 1

*Rank*	Drug	Condition (Reference)	Classification/Use
*1*	Metampicillin		Antibiotic
*2*	Vancomycin	Asthma* (14)	Antibiotic
*3*	Diazoxide		Vasodilator
*4*	Alverine		Spasmolytic
*5*	PF-01378883-00		Unknown
*6*	Felbinac		Anti-inflammatory
*7*	SR-95639A		Unknown
*8*	Zaprinast	Bronchoconstriction (6)	Phosphodiesterase inhibitor
*9*	Trichostatin A	Hyperresponsiveness (3)	Histone deacetylase inhibitor
*10*	Metamizole sodium	Asthma* (39)	Analgesic
*11*	Meprylcaine		Local anesthetic
*12*	PF-00562151-00		Unknown
*13*	Trichostatin A	Hyperresponsiveness (3)	Histone deacetylase inhibitor
*14*	Tetroquinone		Chemical compound
*15*	LY-294002	Hyperresponsiveness (22)	Phosphatidylinositol 3-kinase inhibitor
*16*	Chlortetracycline		Antibiotic
*17*	Equilin		Conjugated estrogen
*18*	Sulfabenzamide		Anti-infective
*19*	Yohimbine		Alkaloid
*20*	Rifampicin	Asthma* (21)	Antibiotic
*21*	Tetrandrine	Asthma (99)	Calcium channel blocker
*22*	Dihydrostreptomycin		Antibiotic
*23*	Acyclovir	Asthma (57)	Antiviral
*24*	Fluorometholone		Corticosteroid
*25*	Trichostatin A	Hyperresponsiveness (3)	Histone deacetylase inhibitor

Rank is order of correlation. CMAP, connectivity map.

* Adverse effect.

**Table 2.** CMAP compounds that mimicked signature 2

*Rank*	Drug	Condition (Reference)	Classification/Use
*1*	Arotinoid acid		Arotinoid
*2*	Folic acid	Asthma* (7)	Vitamin
*3*	Levodopa		Dopamine agonist
*4*	Acebutolol		Beta adrenergic receptor blocker
*5*	Alvespimycin		HSP90 inhibitor
*6*	Isoxsuprine	Asthma (mixed) (11, 93)	Beta adrenergic receptor agonist
*7*	PHA-00851261E		Unknown
*8*	Sulfabenzamide		Antibiotic
*9*	Cefaclor		Antibiotic
*10*	Iproniazid	Bronchoconstriction (5)	Antidepressant
*11*	Amitriptyline	Bronchoconstriction (2)	Antidepressant
*12*	Pioglitazone	Asthma (55)	PPAR agonist
*13*	Fluorocurarine		Sympathetic ganglion blocker
*14*	Piperacillin	Bronchoconstriction* (52)	Antibiotic
*15*	Tanespimycin		HSP90 inhibitor
*16*	Dyclonine		Local anesthetic
*17*	Naftifine		Antifungal
*18*	Alpha ergocryptine		Alkaloid
*19*	Paroxetine		Antidepressant
*20*	Josamycin		Antibiotic
*21*	Mecamylamine	Bronchoconstriction (28)	Cholinergic antagonist
*22*	Etanidazole		Decreases glutathione
*23*	Digoxigenin		Steroid
*24*	Medrysone		Corticosteroid
*25*	PF-00539745-00		Unknown

Rank is order of correlation. CMAP, connectivity map; HSP, heat shock protein; PPAR, peroxisome proliferator-activated receptor.

* Adverse effect.

**Table 3.** CMAP compounds that mimicked signature 3

*Rank*	Drug	Condition (Reference)	Classification/Use
*1*	Imipramine		Antidepressant
*2*	Cinchonine		Alkaloid
*3*	Sulmazole	Bronchoconstriction (60)	Adenosine receptor antagonist
*4*	Amrinone	Bronchoconstriction (44)	Phosphodiesterase inhibitor
*5*	Droperidol	Bronchoconstriction (73)	Dopamine antagonist
*6*	Canadine		Alkaloid
*7*	Domperidone	Asthma (37)	Dopamine antagonist
*8*	Naringenin	Asthma (76)	Flavinoid
*9*	Amitriptyline	Bronchoconstriction (2)	Antidepressant
*10*	Tanespimycin		HSP90 inhibitor
*11*	Diethylstilbestrol	Asthma* (97)	Nonsteroidal estrogen
*12*	2-Aminobenzenesulfonamide		Carbonic anhydrase inhibitor
*13*	Harmaline		Alkaloid
*14*	Furosemide	Asthma (48)	Diuretic
*15*	Cefotaxime		Antibiotic
*16*	3-Nitropropionic acid		Mycotoxin
*17*	Furosemide	Asthma (48)	Diuretic
*18*	Boldine		Alkaloid
*19*	Fulvestrant		Chemotherapy
*20*	Levothyroxine sodium	Asthma* (9)	Synthetic thyroxine
*21*	Homatropine		Anticholinergic
*22*	Sulfaquinoxaline		Antiprotazoal
*23*	Terguride		Serotonin antagonist and dopamine agonist
*24*	Naftifine		Antifungal
*25*	Raubasine		Antihypertensive

Rank is order of correlation. CMAP, connectivity map; HSP, heat shock protein.

* Adverse effect.

We also examined the top 25 compounds that reversed each signature. For *signature 1*, we found that ~50% were beneficial in experimental or clinical airway disease (Table 4) (19, 26, 35, 53, 54, 63, 68, 80, 83, 94). Notably, two compounds—isoetarine and flunisolide—were identified as stand-alone or combination therapies for asthma (20, 45). None of the compounds that reversed *signature 1* were found to worsen or induce asthma. Of the compounds that reversed *signature 2*, 13 were beneficial and decreased bronchoconstriction (15, 43, 71) or AHR (3, 43, 49, 55, 67, 68) (Table 5), and one induced asthma (87). For *signature 3*, eight were beneficial and mitigated bronchoconstriction (29, 32) or AHR (3, 22, 26) and decreased asthma symptoms (16, 74) or incidence of asthma (46) (Table 6). An additional two increased the risk of (27) or induced (89) asthma. None of the compounds that reversed *signature 2 or 3* had evidence for clinical use. A 3 × 2 contingency table analysis and a two-tailed χ^2^ test revealed that the chance of identification of a compound that was of potential benefit in asthma/AHR was not different among signatures [χ^2^ (2, *n* = 75) = 2.69, *P* = 0.26]. The performance of a Fisher’s exact test on the number of combined clinical agents identified 0by the vagal ganglia (two of 50) and the number of combined clinical agents identified by the airway samples (zero of 100) revealed a trend in the vagal ganglia to identify a greater number of clinical agents (*P* = 0.10). Thus three important observations arose from these studies: *1*) the vagal ganglion transcriptome was as effective as the airway tissue in identification of potential therapeutics; *2*) clinical agents were only identified by the vagal ganglia transcriptome; and *3*) compounds that mimicked or reversed a signature were both of potential significance in therapeutic discovery (Tables 7 and 8).

**Table 4.** CMAP Compounds that reversed signature 1

*Rank*	Drug	Condition (Reference)	Classification/Use
*6076*	Etofylline	Asthma (94)	Bronchodilator
*6077*	(−)-Atenolol		Beta adrenergic receptor antagonist
*6078*	Mevalolactone		Chemical compound
*6079*	Ornidazole		Antiprotozoal
*6080*	Solasodine		Alkaloid
*6081*	Flunisolide	Asthma* (20)	Glucocorticoid
*6082*	Etacrynic acid	Bronchoconstriction (63)	Diuretic
*6083*	Tanespimycin		HSP90 inhibitor
*6084*	Etamivan	Asthma (83)	Respiratory stimulant
*6085*	Solanine		Alkaloid
*6086*	Procaine	Asthma (80)	Local anesthetic
*6087*	Ciprofloxacin		Antibiotic
*6088*	Valproic acid	Hyperresponsiveness (68)	Anticonvulsant
*6089*	Alvespimycin		HSP90 inhibitor
*6090*	Isoetarine	Asthma* (45)	Bronchodilator
*6091*	Valproic acid	Hyperresponsiveness (68)	Anticonvulsant
*6092*	Wortmannin	Hyperresponsiveness (26)	Phosphatidylinositol 3-kinase inhibitor
*6093*	Furaltadone		Antibiotic
*6094*	Sirolimus	Asthma (54)	Immunosuppressant
*6095*	Oxymetazoline	Asthma (35)	Decongestant
*6096*	Homatropine		Anticholinergic
*6097*	Urapidil	Bronchospasm (53)	Antihypertensive
*6098*	Minocycline	Asthma (19)	Antibiotic
*6099*	Estriol		Estradiol metabolite
*6100*	Spironolactone		Diuretic

Rank is order of correlation. CMAP, connectivity map; HSP, heat shock protein.

* Approved for clinical treatment of asthma.

**Table 5.** CMAP compounds that reversed signature 2

*Rank*	Drug	Condition (Reference)	Classification/Use
*6076*	Amodiaquine	Bronchoconstriction (15)	Antimalarial
*6077*	Sulfachlorpyridazine		Sulfonamide
*6078*	Trichostatin A	Hyperresponsiveness (3)	Histone deacetylase inhibitor
*6079*	Lisuride		Anti-Parkinson reagent
*6080*	Mephenytoin		Anticonvulsant
*6081*	Nalbuphine		Opioid antagonist
*6082*	Acetylsalicylsalicylic acid	Asthma* (87)	Aspirin impurity
*6083*	Tamoxifen	Asthma (67)	Estrogen receptor blocker
*6084*	Pioglitazone	Asthma (55)	PPAR agonist
*6085*	Trichostatin A	Hyperresponsiveness (3)	Histone deacetylase inhibitor
*6086*	Valproic acid	Hyperresponsiveness (68)	Anticonvulsant
*6087*	Bromperidol		Neuroleptic
*6088*	Lomustine		Chemotherapy
*6089*	Midecamycin		Antibiotic
*6090*	Prestwick-664		Unknown
*6091*	Convolamine		Sensory nerve blocker
*6092*	Trichostatin A	Hyperresponsiveness (3)	Histone deacetylase inhibitor
*6093*	Cotinine		Alkaloid
*6094*	Berberine	Bronchoconstriction (71)	Alkaloid
*6095*	Trichostatin A	Hyperresponsiveness (3)	Histone deacetylase inhibitor
*6096*	Naftopidil		Alpha adrenergic blocker
*6097*	Mianserin	Bronchoconstriction (43)	Antidepressant
*6098*	Valproic acid	Hyperresponsiveness (68)	Anticonvulsant
*6099*	Tretinoin	Hyperresponsiveness (49)	All-trans retinoic acid
*6100*	Trichostatin A	Hyperresponsiveness (3)	Histone deacetylase inhibitor

Rank is order of correlation. CMAP, connectivity map; PPAR, peroxisome proliferator-activated receptor.

* Adverse effect.

**Table 6.** CMAP compounds that reversed signature 3

*Rank*	Drug	Condition (Reference)	Classification/Use
*6076*	Cycloserine		Antibiotic
*6077*	Irinotecan		Chemotherapy
*6078*	Wortmannin	Hyperresponsiveness (26)	Phosphatidylinositol 3-kinase inhibitor
*6079*	Thiocolchicoside		Muscle relaxant
*6080*	Perhexiline	Bronchoconstriction (29)	Antianginal
*6081*	Hydroflumethiazide		Diuretic
*6082*	Halcinonide		Corticosteroid
*6083*	Amodiaquine		Antimalarial
*6084*	Sulfafurazole	Asthma (74)	Sulfonamide
*6085*	Colecalciferol	Asthma (46)	Vitamin D
*6086*	Dimethadione		Anticonvulsant
*6087*	Prilocaine		Local anesthetic
*6088*	Paracetamol	Asthma* (27)	Acetaminophen
*6089*	Trichostatin A	Hyperresponsiveness (3)	Histone deacetylase inhibitor
*6090*	Arecoline	Bronchoconstriction* (89)	Alkaloid
*6091*	Isoconazole		Antifungal
*6092*	Sodium phenylbutyrate		Aromatic fatty acid
*6093*	LY-294002	Hyperresponsiveness (22)	Phosphatidylinositol 3-kinase inhibitor
*6094*	Atropine methonitrate	Bronchoconstriction (32)	Anticholinergic; bronchodilator
*6095*	Epiandrosterone		Steroid hormone
*6096*	Cytisine		Alkaloid
*6097*	0173570-0000		Unknown
*6098*	Isocarboxazid		Antidepressant
*6099*	Tolnaftate		Antifungal
*6100*	Pyridoxine	Asthma (16)	Vitamin B

Rank is order of correlation. CMAP, connectivity map.

* Adverse effect.

**Table 7.** Summary of compounds examined in signatures 1–3

	*Signature 1*	*Signature 2*	*Signature 3*
Mimic			
* n*	25	25	25
* *“Beneficial”	7	4	8
* *“Worsen”	3	2	2
* *Mixed	0	1	0
* *Effect unknown	15	18	15
* *Clinical	0	0	0
Reverse			
* n*	25	25	25
* *“Beneficial”	13	13	8
* *“Worsen”	0	1	2
* *Mixed	0	0	0
* *Effect unknown	12	11	15
* *Clinical	2	0	0
Summary			
* n*	50	50	50
* *“Beneficial”	20	17	16
* *“Worsen”	3	3	4
* *Mixed	0	1	0
* *Effect unknown	27	29	30
* *Clinical	2	0	0

**Table 8.** Common classifications of beneficial drugs

Categorization	Drug Name	*Signature*
Phosphodiesterase inhibitor	Zaprinast	*1*
	Amrinone	*2*
Phosphatidylinositol 3-kinase inhibitor	LY-294002	*1, 3*
	Wortmannin	*1, 3*
Bronchodilator	Etofylline	*1*
	Isoetarine	*1*
	Atropine methonitrate	*3*
Diuretic	Etacrynic acid	*1*
	Furosemide	*3*
Anticonvulsant	Valproic acid	*1, 2*
Histone deacetylase inhibitor	Trichostatin A	*1, 2, 3*
Antidepressant	Iproniazid	*2*
	Amitriptyline	*2*
	Paroxetine	*2*
	Mianserin	*2*
	Amitriptyline	*3*
Vitamin or vitamin derivative	Tretinoin	*2*
	Colecalciferol	*3*
	Pyridoxine	*3*
Anticholinergic	Mecamylamine	*2*
	Atropine methonitrate	*3*

#### Some differentially expressed transcripts in both the vagal ganglia and airway are asthma-associated genes.

The finding that *signatures 1–3* identified potential or known therapeutic compounds suggested that each signature might contain transcripts of known significance to asthma. To examine this possibility, we queried DisGeNET, a catalog of genes and variants associated with human disease (59). We also assessed each transcript for associations with nervous system disorders for a comparison.

There were many transcripts not found in the DisGeNET database (Supplemental Tables S4–S6; shown as continuous “///”). For transcripts that were found in the DisGeNET database, ~5.5% (*signature 1*), 12.2% (*signature 2*), and 8.9% (*signature 3*) were categorized as being a biomarker, gene of altered expression, or genetic variant of asthma (Supplemental Tables S4–S9; blue rows). *Signature 1* had the greatest percentage (70.7%) of transcripts associated with nervous system disorders (Supplemental Tables S4 and S7; gray-shaded rows). By comparison, 44.3% of transcripts comprising *signature 2* (Supplemental Tables S5 and S8; gray-shaded rows) and 62.9% of transcripts comprising *signature 3* were associated with nervous system disorders (as a biomarker, gene of altered expression, or genetic variant; Supplemental Tables S6 and S9; gray-shaded rows). Therefore, DisGeNET revealed that all signatures contained asthma-associated genes, with *signature 1* containing the fewest asthma-associated genes and the greatest number of those related to the nervous system.

#### The vagal ganglia transcriptome reveals a compound that behaves like a bronchodilator.

One of the goals of this study was to identify potential asthma therapeutics through vagal ganglia transcriptome profiling. We thought this might be especially important, given that some mainstay therapeutics that directly target inflammation, such as glucocorticoids, are not effective in all people with asthma (65). The transcriptome analysis and connectivity mapping proved useful in this regard; however, we wondered whether any of the compounds identified by *signature 1*, which had not previously been linked to AHR or asthma and not classified as anti-inflammatories, might also have potential benefit.

To examine this possibility, we assessed all 50 *signature 1* compounds and identified 25 that were of potential interest (i.e., those that had not been linked to asthma and were not anti-inflammatories). We then eliminated those that were of unknown classification or not of obvious neural significance (i.e., antibiotics, antiprotozoals, diuretics, estrogen, heat shock protein 90 inhibitor). This left eight candidates (diazoxide, alverine, meprylcaine, yohimbine, atenolol, solasodine, solanine, and homatropine). Of those, only homatropine and alverine citrate were of continued interest, due to their ability to modulate vagal nerve activity (1, 4, 8, 23). Because anticholinergics are currently used in asthma (61), we did not further explore homatropine. Thus we selected alverine citrate, a spasmolytic with a proposed mechanism of action different from traditional smooth muscle relaxants (90), for further investigation.

**Fig. 5.**
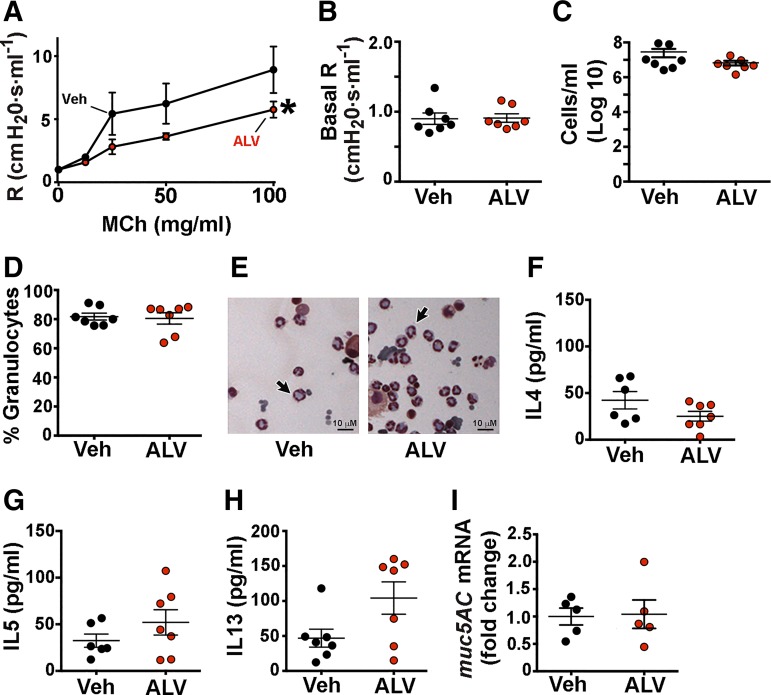
Alverine citrate decreases airway resistance. *A*: airway resistance in wild-type (WT) mice sensitized to ovalbumin (OVA) and treated intraperitoneally with either 15 mg/kg alverine citrate (ALV) or vehicle control (Veh) during the sensitization protocol and then 30 min before the methacholine (MCh) challenge. Resistance (R) was measured after increasing doses of methacholine. Data are means ± SE. For both conditions, *n* = 7 mice. *P < 0.05 by 2-way ANOVA. *B*: baseline airway resistance measured before administration of methacholine. Bronchoalveolar lavage fluid was assayed for total number of cells (*C*) and percentage of granulocyte cells (*D*). *E*: representative hematoxylin and eosin stain of bronchoalveolar lavage fluid from OVA-sensitized WT mice treated with alverine citrate or vehicle control. Arrows indicate examples of granulocytes. Bronchoalveolar lavage concentrations of IL-4 (*F*), IL-5 (*G*), and IL-13 (*H*). *I*: quantitative RT-PCR of *mucin 5AC* (*muc5AC*) mRNA in airways. Individual data points represent data collected from an individual mouse. Bars and whiskers indicate means ± SE.

**Fig. 6.**
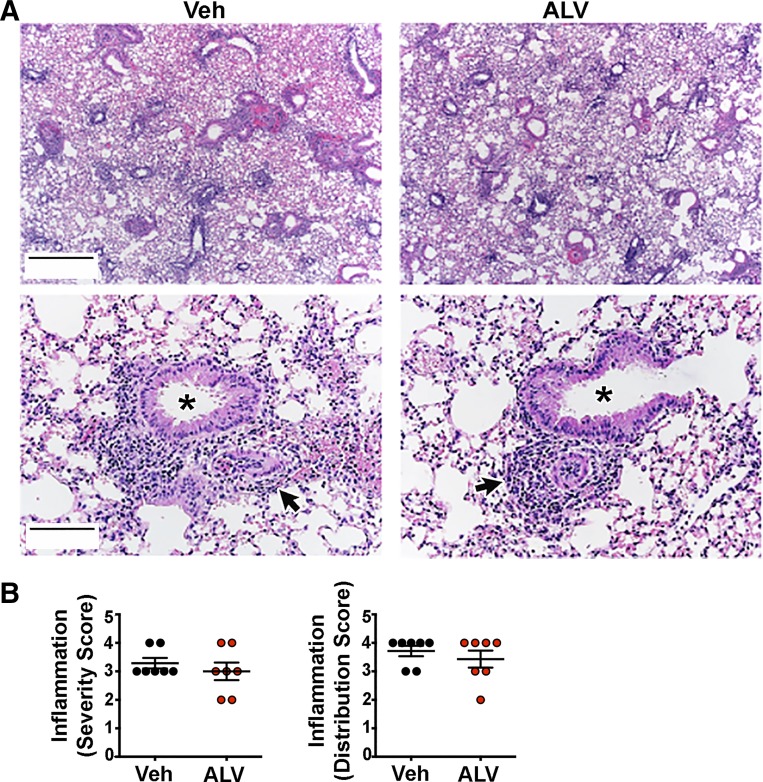
Alverine citrate (ALV) does not decrease bronchoperivascular inflammation in ovalbumin (OVA)-sensitized mice. *A*: representative hematoxylin and eosin staining of mouse lung sections. Asterisks indicate airways; arrows indicate examples of perivascular inflammation. Original scale bars, 700 μm (*top*); 140 μm (*bottom*). *B*: perivascular inflammation score. Individual data points represent data collected from an individual mouse. Data were examined for statistical significance using a nonparametric Mann-Whitney test. Veh, vehicle control.

We treated OVA-sensitized WT mice with alverine citrate using a multiple dosing regimen and found decreased airway resistance in response to methacholine compared with vehicle-treated, OVA-sensitized mice (Fig. 5*A*). This decrease was not likely due to an effect of alverine citrate on baseline airway caliber, as alverine citrate- and vehicle-treated mice had similar basal airway resistance (Fig. 5*B*). We also did not find differences in the number of cells recovered from or the percentage of granulocytes observed in the bronchoalveolar lavage fluid retrieved from alverine citrate-treated and vehicle mice (Fig. 5, *C*–*E*). Bronchoalveolar lavage fluid concentrations of IL-4, IL-5, and IL-13 were also not different between mice that received alverine citrate and vehicle (Fig. 5, *F*–*H*). Although IL-13 levels tended to be higher in alverine citrate-treated mice, this elevation did not reach statistical significance (*P* = 0.094). Since mucus obstruction can increase airway resistance (24), we also examined transcript abundance of *muc5AC*, the major mucus glycoprotein of allergic murine airways (25). We found no effect of alverine citrate (Fig. 5*I*), suggesting that a reduction in mucin expression was not a likely factor mediating alverine citrate’s beneficial effect. The severity and distribution of bronchoperivascular inflammation also did not differ between treatment groups (Fig. 6). We did not test alverine citrate in OVA-sensitized *ASIC1a^−/−^* mice because OVA-sensitized *ASIC1a^−/−^* mice lack AHR.

**Fig. 7.**
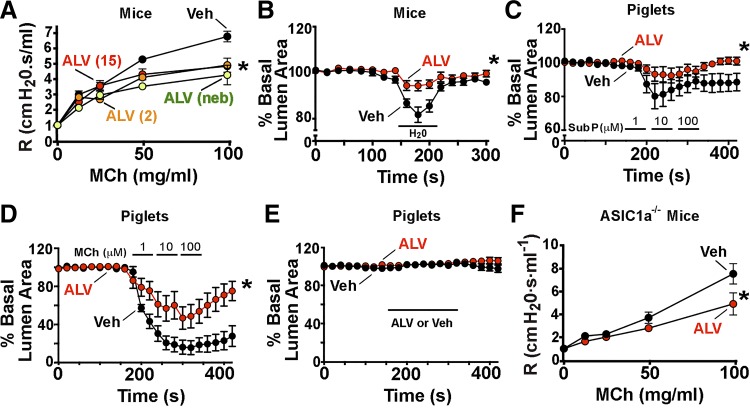
Alverine citrate decreases airway contraction to multiple stimuli and in 2 species. *A*: alverine (ALV; 15 or 2 mg/kg) or vehicle control (Veh) was administered intraperitoneally, 30 min before airway-resistance (R) measurements. Alverine citrate was also nebulized (neb; 15 mg/ml). Data are means ± SE; Veh, *n* = 4 mice; ALV (15 mg/kg ip), *n* = 4 mice; ALV (2 mg/kg ip), *n* = 3 mice; nebulized ALV, *n* = 3 mice. *B*: murine lung slices were incubated with 20 μM alverine citrate or vehicle control and stimulated to contract by perfusion with distilled water for 1 min (marked line, H_2_O). Data are means ± SE of lumen area as a percent of basal area. Veh, *n* = 9; ALV, *n* = 8. *C*: porcine lung slices were incubated with 20 μM alverine citrate or vehicle control and then stimulated to contract by perfusion with increasing concentrations of substance P (Sub P; 1, 10, and 100 μM). Veh, *n* = 5; ALV, *n* = 5. *D*: porcine lung slices were incubated with 20 μM alverine citrate or vehicle control and then stimulated to contract by perfusion with increasing concentrations of methacholine (MCh; 1, 10, and 100 μM). Veh, *n* = 4; ALV, *n* = 5. *E*: porcine lung slices were perfused with 20 μM alverine citrate or vehicle control during the time indicated. No agents were administered to stimulate contraction. Veh, *n* = 5; ALV, *n* = 5. *F*: alverine (15 mg/kg) or vehicle control was given intraperitoneally to nonsensitized *acid-sensing ion channel* (*ASIC*)*1a^−/−^* mice, 30 min before measurement of airway resistance at increasing doses of methacholine. Veh, *n* = 3; ALV, *n* = 3. For all panels, *P < 0.05 for treatment in 2-way ANOVA.

The ascertainment that alverine citrate reduced AHR without affecting inflammation suggested that it might act as a bronchodilator. We confirmed bronchodilator activity in nonsensitized WT mice and found that either acute intraperitoneal or nebulized delivery of alverine citrate decreased airway resistance (Fig. 7*A*). Similar effects were observed when alverine citrate was tested in porcine and murine lung slices. In murine lung slices, alverine citrate blunted water-mediated bronchoconstriction (Fig. 7*B*). In porcine lung slices, alverine citrate acutely reduced substance P- and methacholine-mediated airway contraction. Alverine citrate did not relax porcine airways in the absence of a procontractile stimulus (Fig. 7, *C*–*E*). We also tested whether the actions of alverine citrate were independent of ASIC1a. To do this, we delivered intraperitoneal alverine citrate acutely to nonsensitized *ASIC1a^−/−^* mice and found it reduced airway resistance (Fig. 7*F*). Thus the bronchodilator effect of alverine citrate did not require ASIC1a. Moreover, these findings suggested that other compounds identified by *signature 1* might also have potential therapeutic value.

## DISCUSSION

In the current study, we developed transcriptome signatures from the vagal ganglia of nonsensitized and OVA-sensitized WT and *ASIC1a^−/−^* mice to identify potential therapeutics for AHR and asthma. By treating OVA sensitization and loss of ASIC1a as “interventions,” we identified a subset of transcripts that were responsive to OVA sensitization in the vagal ganglia of WT mice, which upon loss of ASIC1a, were normalized (*signature 1*). For comparison, we developed signatures from previously published transcriptomes of OVA-sensitized WT mouse lung tissues (*signature 2*) (12) and from endobronchial biopsies of human asthmatics (*signature 3*) (100). The use of those transcript signatures to query CMAP revealed many compounds that have already been investigated for AHR and some that are currently used as asthma therapeutics. Of particular interest, only *signature 1*, which was derived from vagal ganglia, identified two clinical agents used to treat asthma (flunisolide, isoetarine). Thus transcriptome profiling of vagal ganglia might be a novel means to discover potential asthma therapeutics.

In our study, we hypothesized that the probing of the vagal ganglia transcriptome might reveal agents important for alleviating AHR with mechanisms independent of dampening inflammation. Indeed, we were able to find several agents. It is possible that the airway tissues used in our study were dominated by inflammatory-induced changes, whereas the vagal ganglia were not. In this case, one might predict that a greater number of beneficial agents, independent of inflammation, would be detected by the vagal ganglia transcriptome compared with the airway tissues. Thus although our study was not designed to answer this question, it is possible that tissue responses to inflammation ultimately impacted the types of drugs identified by each transcriptome.

Many of the compounds identified by *signature 1* were beneficial, independent of whether they mimicked or reversed *signature 1*. This finding suggests that the majority of transcripts comprising *signature 1* were likely compensatory and/or potentially protective in nature. Indeed, previous studies have demonstrated that a sublethal stressor in the nervous system can produce a “damage refractory” state that protects the nervous system from subsequent injury (preconditioning and tolerance) (38, 78, 84). In rats preconditioned with brief seizures, the transcriptional response of the hippocampus to a subsequent seizure is characterized by a neuroprotective suppression of Ca^2+^ signaling (38). Thus it is possible that the vagal ganglia display a similar preconditioning effect during the antigen-exposure process. If true, then perhaps the examination of the temporal pattern of changes in gene expression, as opposed to a single time point, might provide additional insight.

The DisGeNET analysis revealed asthma-associated genes in each signature. Only one gene, *solute carrier family 26, member 4*, overlapped between *signatures 2* and *3*. All other asthma-associated genes were unique across signatures. Given this distinct pattern, it is interesting to speculate whether *signatures 1–3* contain a unique, predictive value regarding asthma symptoms and/or response to asthma therapeutics. It is also interesting to consider whether some genes comprising each signature, that are not currently associated with asthma, might be new asthma candidate genes and/or biomarkers.

We identified alverine citrate as a bronchodilator that reduced airway constriction and decreased airway resistance. Alverine citrate is approved in Europe and other countries for treating irritable bowel syndrome (98). Earlier reports indicated that alverine citrate is not a narcotic or anticholinergic (70, 92). Interestingly, subsequent studies determined that alverine citrate blocks Ca^2+^ entry into smooth muscle and neurons (8). Two additional reports found that alverine citrate blocked action potentials and reduced vagal sensory nerve hyperexcitability (1, 23). We speculate that alverine citrate’s prevention of bronchoconstriction likely involves vagal neurons and/or airway smooth muscle, probably by inhibiting Ca^2+^ channels. It is also interesting to consider the potential benefit of alverine citrate for people with asthma. Alverine citrate is prescribed for nonasthmatic conditions, is a readily available oral agent, and has a good safety profile. Although no data are presently available to evaluate whether alverine citrate modifies AHR in humans, we speculate that it, or perhaps its derivatives, may provide relief and have a beneficial effect in some people with asthma. Perhaps those that are resistant to anti-inflammatory therapy would benefit most.

A potential limitation of our study is that we examined the entire vagal ganglia, including any satellite cells, blood cells, collagen cells, and/or immune cells (42) that might be present at the time of ganglia dissection. However, this approach allowed for the probing of the entire ganglia and was more similar to strategies used to assess transcriptomes of airway tissues (i.e., mixed cell types) (12, 100). The presumed diversity of cells present in the ganglia at the time of dissection might also explain the vast array of compounds that were identified by the vagal ganglia transcriptome. We also recognize that by not querying airway-specific vagal neurons, some signal detected in the vagal ganglia might have arisen from esophageal sensitization and not airway sensitization (101). Thus whereas single-cell RNA sequencing has challenges that can diminish information obtained (36, 69), it is possible that the performance of single-cell RNA sequencing on pulmonary-innervating neurons might provide neuron-specific transcripts important for AHR and eliminate potential noise (96).

It is also important to note that *signatures 1* and *2* were derived from mice, whereas CMAP is a collection of gene signatures derived from human cell lines (41). Yet even with species differences, we were able to identify two clinical drugs for treating asthma using *signature 1*. Others have also found beneficial the use of mouse data to query CMAP for human conditions (33). Another consideration is that the cell lines in CMAP were cancer cell lines (41). Perhaps a more useful data set would be one derived from a panel of drugs applied to vagal ganglia neurons; however, to the best of our knowledge, such a data set does not exist.

We used *ASIC1a^−/−^* mice as a tool to identify potential therapeutics. Yet it is possible that the involvement of nerves in asthma may be via distinct receptors and mechanisms that were not considered in the current study (10, 88, 91, 96). An additional consideration is that whereas OVA sensitization is a good model for allergic inflammation, it might not fully recapitulate the human condition of asthma (77). However, the use of transcripts derived from endobronchial biopsies of human asthmatics (100) to query CMAP did not yield any clinical agents useful for treating asthma, whereas use of transcripts derived from the OVA-sensitization model did. Therefore, whereas care should be taken in extrapolating our findings to a clinical perspective, our data suggest that the OVA-sensitization model can provide meaningful and clinically relevant information regarding asthma therapeutics.

Finally, we identified several additional compounds, not previously linked to asthma and not yet tested in airway disease. Perhaps some of them might be useful for attenuating AHR and treating asthma or other airway diseases. It is also possible that the adaption of a similar strategy that was developed for the vagal ganglia and the application of it to other tissues might reveal unanticipated insight into asthma pathogenesis and potential therapeutics.

## GRANTS

This work was supported, in part, by the National Heart, Lung, and Blood Institute Grants 1K99-HL-119560-01A1 and 4R00-HL-119560-03 (to L. R. Reznikov) and 1P01-HL-091842 (to M. J. Welsh), National Center for Advancing Translational Sciences Grant 10T2TR001983-01 (to L. R. Reznikov), America Asthma Foundation (to D. A. Stoltz), and Roy J. Carver Charitable Trust. M. J. Welsh is an Investigator of the Howard Hughes Medical Institute.

## DISCLOSURES

No conflicts of interest, financial or otherwise, are declared by the authors.

## AUTHOR CONTRIBUTIONS

L.R.R., D.K.M., M.A.A., N.L.B., T.B.B., D.A.S., and M.J.W. conceived and designed research; L.R.R., D.K.M., M.A.A., S.-P.K., Y.-S.J.L., N.L.B., and M.P. performed experiments; L.R.R., N.L.B., and T.B.B. analyzed data; L.R.R., D.K.M., M.A.A., S.-P.K., Y.-S.J.L., N.L.B., T.B.B., and M.J.W. interpreted results of experiments; L.R.R. prepared figures; L.R.R., D.K.M., M.A.A., D.A.S., and M.J.W. drafted manuscript; L.R.R., D.K.M., M.A.A., S.-P.K., Y.-S.J.L., N.L.B., T.B.B., M.P., D.A.S., and M.J.W. edited and revised manuscript; L.R.R., D.K.M., M.A.A., S.-P.K., Y.-S.J.L., N.L.B., T.B.B., M.P., D.A.S., and M.J.W. approved final version of manuscript.

## Supplemental Data

Table S1 Table S1: Transcripts induced or repressed by OVA-sensitization in wild-type and ASIC1a-/- mice - .xlsx (95 KB)

Table S2 Table S2: Transcripts differentially expressed in the vagal ganglia of saline-treated wild-type (WT) and ASIC1a-/- mice - .xlsx (49 KB)

Table S3 Table S3: Transcripts induced or repressed by OVA-sensitization in wild-type (WT) mouse lung or bronchial samples from human asthmatics. Blue indicates transcripts in which Affymetrix identifiers wer - .xlsx (60 KB)

Table S4 Table S4: DisGeNET analysis of Signature #1 - .xlsx (17 KB)

Table S5 Table S5: DisGeNET analysis of Signature #2 - .xlsx (15 KB)

Table S6 Table S6: DisGeNET analysis of Signature #3 - .xlsx (11 KB)

Table S7 Table S7: Full DisGeNET analysis of transcripts comprising Signature #1 - .xls (3 MB)

Table S8 Table S8: Full DisGeNET analysis of transcripts comprising Signature #2 - .xls (7 MB)

Table S9 Table S9: Full DisGeNET analysis of transcripts comprising Signature #3 - .xls (4 MB)
